# Food allergy spectrum in the tropic: clinical and epidemiological profiles in a colombian hospital. A cross-sectional study

**DOI:** 10.3389/fimmu.2023.1291275

**Published:** 2023-12-19

**Authors:** Manuela Olaya-Hernandez, Laura Del Mar Vasquez, Diana Lucia Silva, Sofia Martinez-Betancur, Maria Guerra, Oriana Arias, Luis Fernando Ramirez, Carlos Daniel Serrano

**Affiliations:** ^1^ Valle del Lili Foundation, Cali, Colombia; ^2^ Faculty of Health Sciences, ICESI University, Cali, Colombia

**Keywords:** food hypersensitivity, food allergy, immunoglobulin E, food allergy diagnosis, food sensitization, oral food challenges

## Abstract

**Introduction:**

Food allergy affects 2-10% of the general population; it is more frequent among children than among adults, and it is one of the leading causes of anaphylaxis. Diagnosis of food allergy requires a detailed medical history, skin tests, specific immunoglobulin E (IgE) tests for the food involved, and an oral challenge as final confirmation.

**Objectives:**

This study aimed to describe the clinical and epidemiological characteristics of patients who underwent oral food challenges for suspected food allergies in a reference center in Colombia.

**Methodology:**

An observational, descriptive, cross-sectional and retrospective study was conducted. Data were retrospectively collected from patients who were evaluated in the allergology service and suspected of food allergy from 2011 to 2018. Quantitative variables are presented as means or medians depending on the normality of the distribution (assessed by the Shapiro-Wilk test), and categorical variables are presented as frequencies and percentages.

**Results:**

A total of 215 controlled open challenges were performed on 176 patients, most of whom were children (69%). Thirty-one patients (17%) required another oral challenge with a second food, and 11 (6.25%) required another oral challenge with three foods. Twelve oral challenges (5.58%) were positive. Of these, five challenges were positive for cow’s milk, 5 were positive for shrimp, and 2 were positive for legumes (peanuts and lentils).

**Conclusion:**

The frequency of confirmed food allergies and the profile of food allergies in our population differs from that reported in other parts of the world.

## Introduction

Food allergy is an adverse immune response to food proteins (1). It can be categorized into two types based on its pathophysiological mechanism: immunoglobulin E (IgE)-mediated food allergy, in which clinical symptoms generally appear immediately (5 min -1 hour) after ingesting food; and non-IgE-mediated food allergy, in which clinical symptoms are delayed (usually more than 4 hours after food intake) (2).

IgE-mediated food allergies affect 2-10% of the general population and are more frequent in children than in adults (3). The prevalence may vary depending on the region and the foods most frequently involved (4). The prevalence of non-IgE-mediated food allergies has rarely been studied. Furthermore, few studies have examined the prevalence of food allergies in Latin America and Colombia.

Sánchez and Sánchez (5) reported that there was variability objectives, concepts, age groups, and evaluation methodology of studies that examine food allergies in Latin America; therefore, they were unable to accurately determine the prevalence in the region. However, they observed a higher frequency of allergy to fruits and vegetables in Latin American than in Europe and the United States (5).

In Colombia, sensitization to foods typical of the region, such as yellow potatoes, guavas, bananas, and mangos, has been reported. However, few reports have evaluated whether this sensitization corresponds to a true food allergy (5).

To confirm a diagnosis of food allergy, it is necessary to carry out a detailed clinical history, skin tests and IgE tests for the suspected food to identify the presence of sensitization. When the involvement cannot be determined, performing an oral challenge with the food in question is necessary to confirm or rule out the presence of an allergy (6).

During the challenge, portions of the suspected food are gradually administered to the patient with progressive dose increases until a predetermined final dose is reached and the presence of allergy-related signs and symptoms is evaluated. Two strategies have been described to perform challenges with food: 1) double-anonymized placebo-controlled challenge, which is more commonly used in the research setting, and 2) simple masked or open challenge, which is the most commonly used strategy in clinical practice (7, 8).

An oral challenge is positive when the patient presents signs and symptoms consistent with an allergic reaction, which may affect different systems: urticaria/angioedema and pruritus (skin); dyspnea, wheezing, and nasal congestion (respiratory system); vomiting, diarrhea, and abdominal pain (gastrointestinal system); hypotension, syncope, and tachycardia (cardiovascular system); or drowsiness and lethargy (neurological system) (8).

Given that few studies have examined food allergy confirmed by oral challenge in Latin America and Colombia, this study aims to describe the clinical and epidemiological characteristics of patients who underwent oral challenge for suspected food allergy in a reference hospital in Colombia.

## Methods

In this cross-sectional descriptive observational study, information was collected from the medical records at Fundación Valle de Lili between 2011 and 2018. Patients who were evaluated in the Allergology Service with suspected food allergies and who underwent an oral challenge with the foods involved were included in this study. Patients with incomplete medical records were excluded.

Demographic and clinical data such as clinical presentation, sensitization to food and aeroallergens confirmed by skin tests and IgE tests, history of allergic disease, and results of oral challenge were collected.

The participants were divided into five groups according to the food involved: 1) shellfish (patients who reported symptoms after eating shrimp, prawn, octopus, clam, squid, crab, or lobster); 2) legumes (those who reported symptoms after eating lentils, soybeans orpeanuts); 3) nuts (those who reported symptoms after eating walnuts, almonds, hazelnuts, macadamia nuts, and sesame seeds); 4) fruits (those who reported symptoms after eating strawberry, tomato, or pineapple); and 5) cereals (those that reported symptoms after eating wheat).

Quantitative variables are presented as means or medians depending on the normality of the distribution (assessed by the Shapiro-Wilk test), and categorical variables are presented as frequencies and percentages.

The oral food challenges conducted at our center follow a standardized protocol based on the specific food allergen being tested. These challenges are conducted at 30-minute intervals, with vital signs assessed before each dose. The age criteria for blind challenges are four years for cow’s milk and two years for eggs.

The cow’s milk challenge involves the use of whole milk, with ascending doses as follows: 2-5-10-25-50-100-150 ml.

For egg challenges, we do it with boiled eggs, starting with the yolk at 1/8, 1/4, and 1/2 portions. If tolerated, we proceed with the egg white, also at 1/8, 1/4, and 1/2 portions.

In the case of shrimp challenges, we use shrimp cooked solely with salt, with doses beginning at 5-20-60 grams.

For challenges involving nuts, including lentils for standardization, testing is begging with doses administered at 5-15-45 grams.

Following each provocation, patients are required to undergo a two-hour observation period in our allergology service, during which clinical monitoring and vital sign assessments are conducted.

Specific IgE tests are carried out using Thermofisher’s ImmunoCap on the Phadia 100 system, with results considered positive if they exceed the cutoff value of 0.35 kUA/L.

Skin test, also known as Prick Test, are conducted using standardized allergens provided by Inmunoteck, which are standardized based on proteins at 10 mcg/1ml, considered positive as a wheal diametes exceeding 3 mm.

These tests are performed on the anterior forearm. The procedure begins with cleaning the area using gauze and alcohol. Subsequently, the skin is marked with a fine-tipped marker adjacent to where the drops of allergenic extracts will be applied. A single drop of the allergenic extract under evaluation is placed on the pre-marked area of the skin. Using a lancet, the drop is punctured for approximately one second at a perpendicular angle of 90° to the skin, allowing a small amount of the solution to penetrate into the epidermis. The results are typically read after a 15-20 minute waiting period. Initially, the nurse assesses and provides the preliminary result, which is then confirmed by the attending doctor.

The research complied with current regulations on bioethical research and obtained the authorization of the ethics committee of the institution “Biomedical Research Ethics Committee of the “Fundación Valle del Lili IRB” and due to the design of the study it was not necessary to fill out informed consent.

## Results

Three hundred eighteen patients were considered eligible, and 176 met the inclusion criteria. Of these, 215 open oral challenges were performed. Most patients were younger than 18 years old (69%), with a median age of two years (1-6). In adults, the median age was 43.5 (29-58). A total of 57.3% of those under 18 and 31.4% of adults were male. Regarding comorbidities, among children under 18 years of age, the prevalence rates of rhinoconjunctivitis, asthma and atopic dermatitis were 37.7%, 19.6% and 31.9%, respectively, while in adults, the rates were 48.1%, 16.4% and 3.2%, respectively. Urticaria was reported in 16.6% of adults (**Table 1**).

**Table 1:** General characteristics of the population.

	Pediatric n=122 (%)	Adults n=54 (%)
Age (RIC)	2 (1-6)	43.5 (29-58)
Gender		
Male	70 (57.3)	17 (31.4)
Female	52 (42.6)	37 (68.5)
Personal history		
Rhinoconjunctivitis	46 (37.7)	26 (48.1)
Asthma	24 (19.6)	5 (16.4)
Atopic dermatitis	39 (31.9)	2 (3.7)
Urticaria	7 (5.7)	9 (16.6)
Allergy to hymenoptera	1 (0.8)	1 (0.8)
Drug allergy	5 (4.1)	4 (7.41)
Family history of allergy	67 (58.2)	14 (35.9)

Skin tests with food were performed in 62 children (50%) and 34 adults (62%), and sensitization was found in 27.4% and 2.5% of the participants, respectively. The food with the highest frequency of sensitization was eggs (22 patients, 31.88%), followed by shellfish, cow’s milk, and nuts (peanuts, pistachios) (**Table 2**). In those who underwent IgE tests for food, 47 patients (26.7%) were found to be sensitive to cow’s milk, 40 (22.7%) were found to be sensitive to eggs, and 28 (15.91%) were found to be sensitive to shellfish (**Table 2**).

**Table 2:** Sensitization by skin tests and specific IgE to food.

Food	Skin tests n (%)	Specific IgE n (%)
Cow milk	10 ( 5.68)	47 (26.7)
Egg	22 (31.88)	40 (22.73)
Nuts	7 (3.98)	1 (0.57)
Legumes	1 (0.57)	11 (6.25)
Fish	6 (3.41)	16 (9.09)
Seafood	20 (11.36)	28 (15.91)
Fruit	4 (2.28)	6 (4)
Chicken	2 (1.14)	–
Pig	2 (1.14)	–
Pepper	1 (0.57)	–
Veal	–	1 (0.57)

In 86 children (70%) and 40 adults (74%), a prick test was performed with aeroallergens; the results were positive in 47 children (45.6%) and 30 adults (62.5%). The main sensitizers in the pediatric and adult populations were mites in 32 children (58.0%) and 26 adults (86.6%), pollens in 6 children (13.0%) and 10 adults (34.48%), fungi in 1 child (2.17%) and 3 adults (10.3%), cockroach in 7 children (15.5%) and 16 adults (55.1%), dog in 10 children (21.7%) and 8 adults (27.5%), cat in 5 children (10.8%) and 6 adults (20.69%). Additionally, the main sensitizer was latex in 1 individual in the general population (4.0%).

A total of 176 patients were given open food challenges to confirm the diagnosis of their allergy food. 31 required oral challenge with a second food and 11 with three food. In total, only 12 of the 215 challenges were positive (5.58%): 5 challenges were positive for cow’s milk, 5 challenges were positive for shellfish (shrimp) and 2 challenges were positive for legumes (peanuts and lentils) (**Table 3**). Nine patients (75%) had positive skin tests and IgE tests for the implicated food.

**Table 3:** Oral food challenge.

Food implicated	Provocations made	
	Total (n=215) (%)	Positive (n=12) (%)
Cow milk	67 (40.73)	5 (42)
Seafood	58 (68.71)	5 (42)
Egg	36 (41.8)	0
Fish	26 (33.11)	0
Legumes	15 (18.83)	2 (17)
Fruit	7 (6.23)	0
Meat	2 (1)	0
Cereals	1 (3.23)	0
Alcohol	1 (0.5)	0
Jelly	1 (1.85)	0
Sesame	1 (1.85)	0

## Discussion

This study describes the demographic and clinical characteristics, sensitization, and oral challenge to food in 122 children and 54 adults. The presence of more pediatric patients coincides with the findings described in the literature and in several previous studies (4, 9, 10). Regarding gender, men were more commonly affected in the pediatric population, while women were more commonly affected in the adult population (11, 12).

The median age of those under 18 who attended the consultation in this study was two years, coinciding with a historical cohort study conducted in the United States by Willits et al. (13), in which it was also evidenced that atopic dermatitis in the pediatric population was a very frequent comorbidity of food allergy, as occurred in the present study. In the present work, a significant difference was observed between the pediatric and adult populations regarding the history of atopic dermatitis (31.9% vs. 3.2%) and food sensitization (27.4% vs. 2.5%).

The presence of other allergic diseases has a significant impact on quality of life, as well as on direct and indirect costs (14). In this study, apart from atopic dermatitis, rhinoconjunctivitis and asthma were frequently found, consistent with their high prevalence worldwide (14).

Patients were included based on information provided by the parents (children) and self-report information (adults). Those who met the clinical criteria were studied with skin tests and IgE tests, and according to the results and the individual analysis of the case carried out by the specialist, decisions were made about whether to perform an oral challenge with the suspected food (15).

Regarding skin sensitization to food, it was found that the most common sensitizers were eggs, followed by shrimp, prawns, squid, and cow’s milk (16).

In developed countries, the most common food that caused allergic sensitization in the population over six years of age was peanuts (7.6%), followed by shrimp (5.9%), cow’s milk (4.8%), and eggs (3.4%). In these same countries, the most common foods involved in the population aged 1 to 5 years were cow’s milk (21.8%), eggs (14.2%) and peanuts (6.8%) (17). A study carried out in Mexico by Ruiz Segura et al. (18) found that the most common sensitizers among children ≤5 years of age were cow’s milk and eggs, while peanuts, almonds, wheat, soy, corn, shrimp, and kiwi were the most common sensitizers among older children, and apple was the most common sensitizer among adults (18). This differs from the results of the present study since a high frequency of sensitization or allergy to peanuts was not observed herein.

In our services, we conduct skin prick tests using standardized allergens provided by Inmunotek. Specifically, the extracts used for egg white and protein testing are derived from boiled eggs.

Regarding the higher prevalence of sensitization observed in specific IgE or skin prick tests in egg compared to the absence of positive oral challenges, it is worth considering that these patients exhibited low levels of specific IgE or skin prick test results with small diameters. In both cases, these values fell below the cutoff points commonly established in the literature for provocation testing.

Given that this study is retrospective, it begins with oral challenge as the starting point and subsequently assesses sensitization and clinical symptoms. This approach may explain why some patients with cutoff points above the established thresholds did not undergo oral provocations. This limitation represents one of the key challenges of the study.

Some patients required more than one challenge to a different food, which explains the higher number of challenges performed compared to the number of participants. In this study, the positivity rate of the challenges was 5%, which is somewhat higher than that reported in other studies in which the positivity rate is less than 2% (15).

**Figure 1:**
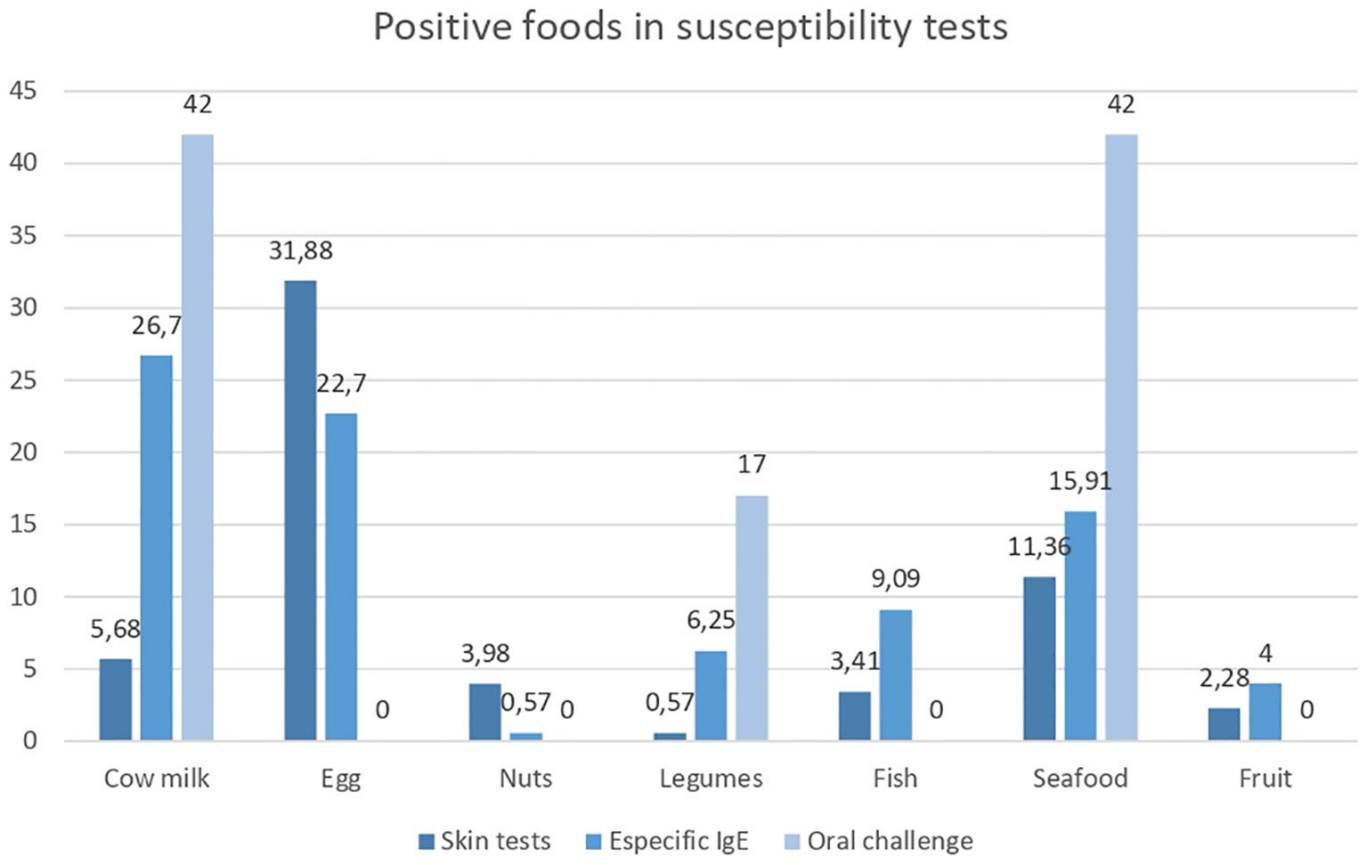
Positive foods in susceptibility tests. Percentage of positive foods in susceptibility tests. **Source:** graph of realization and own authorship.

Of the 215 oral challenges performed, only 12 were positive (5.5%). The foods that induced the best positivity were cow’s milk and shrimp (42% of positive challenges each). The other two positive challenges were with legumes (one with peanuts and one with lentils) (18) (**Figure 1**). Seventy-five percent of the patients with a positive oral challenge test had positive skin tests and specific IgE tests in addition to clinical suspicion, which strengthens the importance of controlled oral challenge with the suspected food for confirming the diagnosis of food allergy.

Remarkably, during the time of the study, even though our country had a high fruit consumption rate, we did not encounter any patients exhibiting suspicion, sensitization, or allergies to tropical fruits. However, over the past year, we have observed several cases, particularly related to bananas. Regrettably, these cases fall outside the timeframe covered by this manuscript’s study period.

The limitation of this study lies in taking as the starting point all the patients taken for the oral challenge and, from there, determining the allergen sensitization tests that were previously performed. The study does not have the possibility of determining what happened to patients with suspected food allergies with allergy tests above the cutoff points for the challenge.

## Conclusion

The allergy profile observed in this study differs from the series reported in countries from other geographic regions. Prospective studies are required to more precisely define the clinical and epidemiological profile of food allergy in tropical countries and other specific aspects related to diet and exposure.

## Data availability statement

The original contributions presented in the study are included in the article/**Supplementary Material**. Further inquiries can be directed to the corresponding author.

## Ethics statement

The studies involving humans were approved by institution “Biomedical Research Ethics Committee of the Fundación Valle del Lili IRB”. The studies were conducted in accordance with the local legislation and institutional requirements. Written informed consent for participation was not required from the participants or the participants’ legal guardians/next of kin in accordance with the national legislation and institutional requirements.

## Author contributions

MH: Conceptualization, Data curation, Investigation, Supervision, Validation, Visualization, Writing – original draft, Writing – review & editing. LV: Conceptualization, Data curation, Methodology, Software, Writing – original draft. DS: Formal Analysis, Supervision, Validation, Visualization, Writing – review & editing. SB: Conceptualization, Formal Analysis, Investigation, Writing – original draft, Writing – review & editing. MG: Conceptualization, Data curation, Investigation, Project administration, Writing – original draft. OA: Data curation, Formal Analysis, Methodology, Project administration, Software, Supervision, Writing – review & editing. LR: Supervision, Visualization, Writing – review & editing. CS: Conceptualization, Formal Analysis, Investigation, Supervision, Validation, Visualization, Writing – review & editing.
